# Retraction Note: Extracorporeal membrane oxygenation support for SARS-CoV-2: a multi-centered, prospective, observational study in critically ill 92 patients in Saudi Arabia

**DOI:** 10.1186/s40001-024-02243-2

**Published:** 2024-12-30

**Authors:** Saad Alhumaid, Abbas Al Mutair, Header A. Alghazal, Ali J. Alhaddad, Hassan Al-Helal, Sadiq A. Al Salman, Jalal Alali, Sana Almahmoud, Zulfa M. Alhejy, Ahmad A. Albagshi, Javed Muhammad, Amjad Khan, Tarek Sulaiman, Maha Al-Mozaini, Kuldeep Dhama, Jaffar A. Al-Tawfiq, Ali A. Rabaan

**Affiliations:** 1 Administration of Pharmaceutical Care, Al-Ahsa Health Cluster, Ministry of Health, 31982 Al-Ahsa, Saudi Arabia; 2 Research Center, Almoosa Specialist Hospital, Al-Ahsa, Saudi Arabia; 3 College of Nursing, Princess Norah Bint Abdul Rahman University, Riyadh, Saudi Arabia; 4 School of Nursing, Wollongong University, Wollongong, Australia; 5 Microbiology Laboratory, Prince Saud Bin Jalawi Hospital, Al-Ahsa, Saudi Arabia; 6 Microbiology Department, Omran General Hospital, Al-Ahsa, Saudi Arabia; 7 Division of Laboratory, Medical Microbiology Department, Maternity and Children Hospital, Al-Ahsa, Saudi Arabia; 8 Division of Neurology, Internal Medicine Department, King Fahad Hofuf Hospital, Ministry of Health, Al-Ahsa, Saudi Arabia; 9 Internal Medicine Department, King Fahad Hofuf Hospital, Ministry of Health, Al-Ahsa, Saudi Arabia; 10 Department of Nursing Education, College of Nursing, Imam Abdulrahman Bin Faisal University, King Faisal Road, Dammam, Saudi Arabia; 11 Department of Microbiology, The University of Haripur, Haripur, 22620 Khyber Pakhtunkhwa Pakistan; 12 Department of Public Health/Nutrition, The University of Haripur, Haripur, 22610 Pakistan; 13 Infectious Diseases Section, Medical Specialties Department, King Fahad Medical City, Riyadh, Saudi Arabia; 14 Immunocompromised Host Research Unit, Department of Infection and Immunity, King Faisal Specialist Hospital and Research Center, 11211 Riyadh, Saudi Arabia; 15 Division of Pathology, ICAR-Indian Veterinary Research Institute, Izatnagar, Bareilly, 243122 Uttar Pradesh India; 16 Infectious Disease Unit, Specialty Internal Medicine, Johns Hopkins Aramco Healthcare, Dhahran, Saudi Arabia; 17 Department of Medicine, Indiana University School of Medicine, Indianapolis, IN USA; 18 Department of Medicine, Johns Hopkins University School of Medicine, Baltimore, MD USA; 19 Molecular Diagnostics Laboratory, Johns Hopkins Aramco Healthcare, Dhahran, Saudi Arabia; 20 Department of Public Health and Nutrition, The University of Haripur, Haripur, 22610 Pakistan

**Retraction Note: European Journal of Medical Research (2021) 26:141** 10.1186/s40001-021-00618-3

The Editor-in-Chief has retracted this article. After publication concerns were raised about the methodology of the study reported. The Editor-in-Chief no longer has confidence in the results and conclusions presented. All authors disagree with this retraction.

